# Integrated Nematode Management Strategies: Optimization of Combined Nematicidal and Multi-Functional Inputs

**DOI:** 10.3390/plants14071004

**Published:** 2025-03-23

**Authors:** Mahfouz M. M. Abd-Elgawad

**Affiliations:** Plant Pathology Department, National Research Centre, El-Behooth St., Dokki 12622, Egypt; mahfouzian2000@yahoo.com

**Keywords:** antagonism, biocontrol, mechanisms, nematode management, additive effect, synergism, optimized strategies

## Abstract

Considerable losses are inflicted by plant-parasitic nematodes (PPNs) due to their obligate parasitism; serious damage occurs in many susceptible crops, and the parasites have a broad distribution worldwide. As most PPNs have a subterranean nature, the complexity of soils in the plant rhizosphere and the structures and functions of the soil food webs necessitate a grasp of the relevant biotic/abiotic factors in order to ensure their effective control. Such factors frequently lead to the inconsistent performance and untapped activity of applied bionematicides, hindering efforts to develop reliable ones. Research efforts that take these factors into account to back the usage of these bionematicides by combining the disease-suppressive activities of two or more agricultural inputs are highlighted herein. These combinations should be designed to boost useful colonization in the rhizosphere, persistent expression of desirable traits under a wide range of soil settings, and/or antagonism to a larger number of plant pests/pathogens relative to individual applications. Relevant ecological/biological bases with specific settings for effective PPN management are exemplified. Determining the relative sensitivity or incompatibility of some biologicals entails studying their combinations and reactions. Such studies, as suggested herein, should be conducted on a case-by-case basis to avoid unsatisfactory outputs. These studies will enable us to accurately define certain outputs, namely, the synergistic, additive, neutral, and antagonistic interactions among the inputs. In optimizing the efficiencies of these inputs, researchers should consider their multi-functionality and metabolic complementarity. Despite previous research, the market currently lacks these types of safe and effective products. Hence, further explorations of novel integrated pest management plans that boost synergy and coverage to control multiple pathogens/pests on a single crop are required. Also, setting economic incentives and utilizing a standardized regulation that examines the authentic risks of biopesticides are still called for in order to ease cost-effective formulation, registration, farmer awareness, and usage worldwide. On the other hand, tank mixing that ensures legality and avoids physical and chemical agro-input-based incompatibilities can also provide superior merits. The end in view is the unraveling of the complexities of interactions engaged with in applying multiple inputs to develop soundly formulated, safe, and effective pesticides. Sophisticated techniques should be incorporated to overcome such complexities/limitations. These techniques would engage microencapsulation, nanopesticides, volatile organic compounds as signals for soil inhabitants, bioinformatics, and RNA-Seq in pesticide development.

## 1. Introduction

Plant-parasitic nematodes (PPNs) are inflicting substantial losses in size and quality for a broad range of key crops. A recent review [1] of losses brought about by PPNs recorded losses of USD 358.24 billion annually for the 40 life-sustaining crops that have substantial value for food and export, i.e., an average of a 13.5% loss in crop production. Traditional and emerging measures for PPN control use a variety of materials and methods that include long-established [2,3,4] or novel [5,6,7,8] chemical nematicides; conventional [9,10,11] or recent, i.e., developed in the last five years, PPN-resistant genotypes [12,13,14]; continuous release of certified seed/planting material [15]; improved soil solarization for the suppression of PPN populations [16]; and novel bionematicides [17]. The utilization of these measures is typically supported by one or more production practices linked directly/indirectly to PPN control. These practices may relate to ploughing, cover crops, crop sequencing, flooding, soil amendments, and/or fallowing. The development of novel materials/techniques is continuously required to address major PPN problems [15,18,19].

These materials/methods generally constitute the bases of current (long-established and still in use) and novel (recently emerged or still in the testing and development phase) strategies for controlling PPNs. Hada et al. [20] confirmed the consensus that none of the present methods possess superiority in all aspects. Certain aspects of optimized PPN control may determine its performance, in terms of being economical, reliable, inclusive, and secure relative to nontargets. Striking examples include various chemicals that can reliably work as nematicides [21] via their contact with nematodes within roots or in the plant rhizosphere [22]. These chemicals can occasionally slow/impede the development of PPN resistance and effectively enhance crop yields but they may also pose health-related and ecological hazards [23]. Therefore, among the ongoing priorities are researching and exploring the synthesis/rational designs of novel chemical and biological nematicides that offer safe, economic, and effective alternatives to unhealthy materials. These approaches may also include avoiding the excessive use of such chemicals via the integration of low doses with compatible synergistic/additive agricultural inputs [23]. Thus, their combinations could potentiate nematode control measures. Furthermore, following these general lines of thinking, three levels in the boosting of PPN management were recently suggested [19]. These included bridging the gap between current and novel strategies for PPN control, using reliable decision support tools and systems in approaching extensive PPN management schemes worldwide, and harmonizing nematicidal operations via the development of additional novel targets. These levels imply, either directly or indirectly, the uses of further combinations that offer potential for a more extensive beneficial microorganism colonization within the plant rhizosphere. The combined materials and techniques are assumed to perform favorably under a broad profile of settings and demonstrate antagonism to a larger number of plant pests/pathogens than would agricultural inputs applied singly [24,25].

Therefore, this review focuses on different aspects of such combined applications. It presents the ongoing advances in the relevant research and techniques. As current agricultural systems are knowledge-intensive regarding these various agricultural inputs, this review highlights related PPN control strategies in general. As the combined effects of nematicidal and multi-functional inputs may greatly vary according to their traits and interactions, bases aiming to boost the outcomes of such diverse combinations are presented herein. Special emphasis is given to combinations that lead to synergistic and additive interactions, along with the necessary settings for optimizing their PPN control processes. Other interactions, both negative and neutral, among the combined inputs are also exemplified. The approaches of advanced techniques are presented to address interactions that can contribute into developing novel strategies for superior PPN management.

## 2. Emerging Strategies Associated with Integrated Nematode Management (INM)

As PPNs wreak havoc on key crops worldwide which endanger agricultural productivity, a large number of strategies have been developed for their control. They may include one or more of a broad variety of organisms known to act as biocontrol agents (BCAs) against PPNs, such as bacteria, fungi, viruses, protists, nematode antagonists, and other invertebrates [26]. Other cultural (e.g., sanitation, flooding, intercropping, fallowing, proper time of sowing and harvesting, fertilizing, chemical nematicides, crop rotation, resistant genotypes, and quarantine) and physical (e.g., heat, soak the seeds in water, electric current, traps, water management, radioactive irradiation, ultrasonic devices, and ultraviolet rays) methods could be engaged as well. Some of these methods and (or) BCAs/their bioactive compounds have, in most cases, been proving viable strategies when applied in combination with minimized doses of chemicals in various scenarios of integrated pest management (IPM) [19,23,27]. In contrast, single/excessive use of these chemicals, especially pesticides, can lead to serious issues such as pest resistance, adverse effects on useful fauna, harmful residues, and other ecological concerns. In other words, emerging combined treatments are trying to represent a safer alternative to traditional methods with more effectiveness against the nematodes. Yet, while setting most of these combinations under the IPM framework can be cost-effective and sustainable techniques to PPN management, a few of them have unfavorable outcomes. Therefore, continuous furnishing of growers, extension workers, and other stakeholders with related information that is necessary for sound PPN management is essential. It can help enhance awareness of the most important PPN pests and diseases. While distinguishing unwanted combinations, this section offers basics and guidance for the most effective control strategies of PPNs, which can also help to optimize the quantity and quality of crop yields.

### 2.1. General Bases of the Emerging INM Strategies

Fundamentally, the use of more than one substance or technique for the purpose of PPN management requires compatibility—not counteracting—between them. This principle should be considered especially for co-application for simultaneous inputs. Sequential application may be used if the efficacy of each material is so separate that its timed usage does not result in incompatible reaction with the other one but rather synergizes or favorably adds to its action. Even for triple and dual-purpose usage of such materials, their combinations should not suppress production/efficacy of nematicidal materials and/or PPN-antagonistic biologicals.

Compellingly, because BCAs are generally more sensitive to many abiotic and biotic factors than nonbiological inputs, relevant settings should be properly deemed. Abiotic factors affecting effectiveness of BCAs include climate as well as physical and chemical composition of the rhizosphere (e.g., soil structure and properties, temperature, and moisture for soil nematodes). Biotic factors engage interactions with nontarget organisms, degree of soil and/or rhizosphere colonization by BCA(s), injury induced by nontarget pathogens/pests, initial population levels of the targeted PPN species, susceptibility of the host plant to the existing species, and host plant species/cultivar [23,24]. Because food webs of soil, where PPNs mostly live, have a cryptic nature, they are largely unknown and need more studies. Surely, biotic/abiotic factors can impose unfavorable outcomes of BCA activity whether individually or combined with other inputs. These may comprise inconsistent (variable) efficacy and slow acting, especially in the context of BCA-field performance. Consequently, they may lead to farmers’ risk aversion and untapped IPM economies [28]. So, it is important to find out more new BCAs/their bioactive compounds and favorably modulate variables that can cause significant improvement in the PPN control potential. Such adjustments should be carried out through conserving or selecting specific settings or BCAs [29,30,31]. For instance, soil type and moisture [32], salinity [33], mulching/integrating organic matter [34], and pH [35] proved to be crucial factors in modifying nematode population levels directly/indirectly by influencing their hosts and/or natural enemies [27,36]. Meyer and Roberts [24] speculated that it is not surprising to find an individual BCA with differential activity in various soil settings. This is due to so many biotic/abiotic factors that affect its activity. In contrast, almost all relevant biocontrol interactions resulting in suppressing PPN diseases can be primarily attributed to one or more of the following general classes of mechanisms exerted by BCAs: competition for resources (e.g., nutrients) and spaces, antibiosis, predation/parasitism, and induced resistance of plants [24]. Surely, there are differential activities of BCAs under various conditions that are also related to specific modes of action. Such mechanisms of beneficial fungal and bacterial nematicides against key PPNs on economically important crops are presented (Table 1). So, their relevant results can still provide useful insights to use them effectively and wisely under the referred settings.

It is also remarkable that, among all PPNs, *Meloidogyne* spp. rank high as the main damaging phytonematodes in many regions of the world. Therefore, their numerous species have often been the major target of INM strategies due to their significant economic and scientific importance. They can live in their natural mode as obligate plant-sedentary endoparasites. However, this does not negate the importance of other PPN genera/species. Following such an assumed importance, the top 10 list documented by a substantial survey [47] is composed of the following genera/species in descending order: (1) RKNs (*Meloidogyne* spp.); (2) cyst nematodes (*Heterodera* and *Globodera* spp.); (3) root lesion nematodes (*Pratylenchus* spp.); (4) the burrowing nematode *Radopholus similis*; (5) *Ditylenchus dipsaci*; (6) the pine wilt nematode *Bursaphelenchus xylophilus*; (7) the reniform nematode *Rotylenchulus reniformis*; (8) *Xiphinema index* (the only virus vector nematode to make the list); (9) *Nacobbus aberrans*; and (10) *Aphelenchoides besseyi*.

We must also take into account all aspects that contribute to reducing the economic cost of any combination used for PPN control strategy. For instance, chemical companies can easily command the transport logistics of *Bacillus*-spp.-based products because their spore forms have a long-lasting shelf life [45]. Unfortunately, the much larger control potential markets frequently sell other BCAs with limited opportunity to withstand or survive the distribution logistics prevalent in the plant protection trade [48]. Hence, adequate formulation technology should always be developed to overcome this impediment, especially when using BCAs with other compatible input(s). Unlike chemical nematicides, which are formulated to boost their transport and effectiveness on the plant, the major parameter in formulating biological agents should be securing their survival and biocontrol activity at high quality. Contrary to the broad spectrum of different additives utilized with chemical pesticides, such substances can mostly interfere with biocontrol activity of BCAs, causing unfavorable effects. Therefore, sophisticated techniques should face such challenges. These techniques could directly/indirectly defeat such difficulties via incorporating micro-encapsulation technology [49], bioinformatics, RNA-Seq in biopesticide evolvement, and considering PPNs spatial distributions [50]. In order to avoid sensitivity of many BCAs to biotic factors, sterile packing has also been an efficient method to prolong the BCAs shelf life, especially for liquid formulations [48].

Moreover, recent calls suggest that it is preferable to bring many such related beneficial microorganisms together under one regulatory framework. Numerous of these microorganisms are applied as fertilizers, seed treatment, in the food industry, soil amendment, silage, and compost additive. Additionally, many developed products containing bacteria or fungi are formally sold as plant growth promoters, plant strengtheners, or soil conditioners. However, because their relevant risks are almost the same—independent from how they are used— experts handling them under one regulation that checks the real risks would smooth their usage in a pragmatic approach for various agricultural systems [23,27,48]. This would certainly facilitate the registration of microbes to manage plant-parasitic nematodes if it were applied. Although there are many small firms that employ cheap labor to produce such microbial products (e.g., bionematicides) at low cost, exclusively for domestic markets, a lot of these products may be efficacious [25]. In numerous developing countries in Latin America, Africa, and Asia, such products are applied only locally to avoid high costs of rigorous registration processes usually demanded in Europe and North America. A standardized regulation that examines the authentic risks would ease their registration and consequent usage worldwide. Moreover, it cuts off costs to end-users, which remain in most markets greater than the alternative nematode management tactics, i.e., chemical nematicides. As a result, current BCA applications against PPNs are frequently relegated to high-value niche markets. Farmers and stakeholders mostly seem reluctant to use novel nematicidal and biological products via paying a premium when there are familiar, easy to utilize, low-cost alternative chemicals. The use of unhealthy nematicides needs to be further minimized by regulations and economic incentives while raising grower’s awareness.

On the other hand, novel chemical nematicides, which are less dangerous for human health and the environment, must be evolved and checked. This is especially for their additive or synergetic effects with other PPN control measure(s) under field conditions. For instance, Dababat et al. [51] urged chemical companies to test any of such promising nematicides on a wide range of wheat germplasms that have different resistance levels against *Heterodera avenae* (the cereal cyst nematode). This will enable examining of whether these nematicides have additive or synergetic effect on the behavior of these wheat *H.-avenae*-resistant genotypes. Another remarkable avenue is to test compatibility of such chemicals with BCAs. This is especially important, as many BCAs are slow to function and often need time to enhance their populations up to the levels desired to be reliable for PPN control. Meanwhile, the growers require alternative methods for PPN management until BCAs can effectively function. Therefore, the sustainability of these BCA-based techniques should be wisely manipulated because the aforementioned complexity of the biotic/abiotic factors is usually linked to agricultural systems. A possible scenario could be to design IPM that is based first on a well-established control method (e.g., a chemically based one) and gradually introduce BCA(s) together with a detailed monitoring scheme in order to cautiously reduce the dependence on chemical control. Such strategies for various agricultural systems should not negate the possible applications of other complementary alternative control techniques. These latter could involve those of the above-mentioned physical and cultural practices that may have a boosting response when combined to one another or/and with BCAs.

### 2.2. Favorable Combinations for Management of PPNs

Many of these combinations contain one or more BCAs due to the current regulations limiting the application of chemical nematicides. Thus, due to their mounting importance, promising BCAs/their bioactive compounds that can synergistically/additively perform against PPNs are presented first herein. Then, other relevant combinations of biologicals with other materials and techniques that provide superior merits for PPN suppression and yield improvement are exemplified as well. These latter may engage other safe agricultural practices and materials without BCAs/their bioactive compounds.

#### 2.2.1. Merits and Considerations for Applying Combined BCAs

Firmly, there are merits of wisely applying more than one BCA in combination in order to involve more merits than single BCA: (i) multiple mechanisms against the target PPN species/race; (ii) capability to adversely affect more than one stage of the life cycle of the intended organism; (iii) various activities of BCAs during the course of the plant-growing season; (iv) enhanced consistency in biocontrol performance over a broad range of soil conditions due to the different ecological niches of the used BCAs; and (v) potential to select different BCAs that adversely affect more than one plant pathogen/pest simultaneously, thus boosting the spectrum of BCA uses [24]. The latter authors gave numerous related practical examples. Such key points for developing and marketing two or more combined BCAs may possibly bring about an effective synergistic/additive interaction. To this end, boosting multi-functionality and stability of combined BCAs could be attained by merging metabolic complementarity with other functional traits (e.g., functional diversity, composition, and redundancy) and obtaining systematical inoculation effects on recipient soils and/or plants [52,53]. In this respect, the ability of BCAs to exploit different resources for their consumption in dynamic metabolic strategies is more likely to result in consolidated and harmonized combination. Also, time-related differences among BCAs in interactions with hosts could lead to synergistic impacts. In these cases, the composition of BCAs can perform biocontrol activity across relatively long time scales. Generally, compounds and cocktails of inoculants or/and BCAs can be based on these foundations to comprise various and beneficial microbes/products. They would preferably serve towards beneficial multi-directional steering of soil ecosystem functions. Normally, the interaction effect on the development of each BCA varies from normal or supported development to exclusion. Main factors that can be manipulated to gain the desired effect rely on traits of BCA and host species/strain, rhizospheric soil microbiome, BCA doses, and the timing of both BCA usage and pest infection. So, further experimentation focusing on these factors can help to find novel eco-friendly PPN control strategies, as there is a dire need for such commercial products. Such combinations may boost the genetic diversity in the plant rhizosphere for sustainable agricultural systems too. Reciprocally, two main concerns should be considered, i.e., testing their possible antagonism and compiling the files of registration for these BCAs together. This might, in turn, increase the product costs [48]. Absolutely, cautious tracing of biotic/abiotic factors to fit the applied BCAs in the targeted settings is a continuous priority.

The ultimate goal of these combinations is to achieve more efficacy in enhancing plant vigor/yield and (or) decreasing PPN population levels or penetration of roots than individual applications [24,54,55,56,57,58,59,60]. Such trends are quite supported by worldwide regulations that ban the application of chemical nematicides, e.g., aldicarb [61] and carbofuran [62]. Hence, it is essential to assure their safety and absorb interaction processes among specific BCAs and/or their bioactive compounds in light of the existing settings. They can pave the way for developing more potent nematicidal products within IPM strategies in both the open fields and greenhouses [27]. However, as protected cultivation aims at adjusting the microclimate to protect crops from unfavorable weather with subsequent year-round cultivation, high yields, improved quality, and/or off-season production, it may also create favorable conditions for PPNs. Therefore, pre-plant fumigation followed by neem cake fortified with BCAs or similar combinations can frequently be applied to suppress high PPN population densities under protected agriculture [63].

Eventually, characterization of distinctive fauna and flora to deal with should always be sought. For instance, some nematodes can feed on fungi and vice versa. The population of fungi in the soil is suppressed if fungivorous nematodes like *Aphelenchus avenae* feed on fungal pathogens of plants. So, *A. avenae* and *Aphelenchoides* spp. caused a reduction in damping of cauliflower due to suppression of *Rhizoctonia solani* [64]. Moreover, *A. avenae* can also suppress reproduction of the nematode *Ditylenchus destructor*. Thus, *A. avenae* has potential as BCAs against both fungi and PPNs. Yet, as *D. destructor* is a plant and fungal feeder, its infestation may be impacted by the plant-associated fungi and suppressed via control of these fungi too [65]. Therefore, successful IPM schemes have to strongly match the attributes of BCA(s) within the context of specific farming operations in order to efficiently fit the existing conditions.

#### 2.2.2. Observing Contradictory Nominations of Some BCAs as Nematicides

Notably, some BCA-related materials may not presently be considered nematicides, though they possess nematode-antagonistic activity. However, it should be emphasized that the future expansion of these materials will help generate large coherent categories that might be still in the process of development, i.e., anticipated nematicides. In this vein, two main and relevant divisions of biostimulants are sourced from plant and microbial origin. Plant biostimulants are frequently reported for a side-suppressive impact on many pests. Biostimulants derived from plants are well documented for an effective suppression of RKN species [66]. Furthermore, a considerable portion of them could form numerous commercial products that are progressively involved in sustainable PPN control strategies. In contrast, the suppressiveness of microbial biostimulants is found largely changeable for PPNs reliable suppression, as related to the crop and to ecological factors. The latter authors recorded several commercial biostimulants derived from plants and possess activity against PPNs. They could also function as rooting, fertilizing, plant defense enhancement, increasing soil beneficial microflora, creation of a nematode-unfavorable environment, repellence, antifeeding, disorientation, and toxicity. Although the related tests indicated the potential usage of biostimulants in PPN management, such results would need to be validated by further experimentation under field conditions. Principally, these authors [66] recommended that various combinations of the tested biostimulants should also be examined for the potential synergism between their different products for RKN control. Likewise, Wilson and Jackson [67] documented that many companies do not list under the bionematicidal section the many products of BCAs that contain bacteria or fungi but are also sold as plant growth promoters, soil conditioners, or plant strengtheners (e.g., many *Trichoderma* spp. products and Agrinos’ proprietary High Yield Technology^TM^ products). These products may improve plants’ ability to withstand PPN invasion, but they are not considered as biological nematicides. Obviously, managing/controlling PPNs by an individual species, strain, or isolate of these biologicals or their bioactive compounds can possibly give effective results [19]. In other words, they are considered as multi-functional inputs. Therefore, favorable synergism among two or more inputs that include these products should further be sought [41,68,69].

#### 2.2.3. Examples of Combined BCAs Alone or with Other Measures for PPN Management

Even compared to chemical nematicides, a consortium of rhizobacteria (*Pseudomonas fluorescens*) and vesicular mycorrhizal fungi (AMF) could be more effective in suppressing PPN populations and controlling root-knot disease in black henbane, *Hyoscyamus niger*, and Egyptian henbane, *H*. *muticus*, two medicinal plant species that were used in experimentation [70]. Yet, the effectiveness of PPN control may vary from one BCA to another. Among BCAs, Khan and Quintanilla [71] considered *Purpureocillium lilacinum*, *Trichoderma* spp., *Pochonia chlamydosporia*, *Aspergillus niger*, *Bacillus* spp., *Pseudomonas fluorescens*, and *Pasteuria* spp. as the most striking examples for effective PPN control. The authors ranked mycorrhizal fungi high too as they are being increasingly explored for PPN management and have proved quite suppressive to many PPN population densities. Surely, their report [71] does not negate the relevant importance of other current and expected BCAs that are still in the pipeline. The combined effect of *Trichoderma harzianum* and *Acremonium strictum* led to great reduction in root-knot disease severity caused by *M*. *incognita* with consequent high tomato yield [72]. In another study, a single BCA could be used in two different formulas. For instance, *Aspergillus niger* F22 producing oxalic acid was utilized in a 1:1 mixture of two wettable powder-type formulations of oxalic acid and *A*. *niger* F22 (spore count, 4.1 × 10^8^ colony-forming units (CFU)/g) at 1000- and 500-fold dilutions to a watermelon field to minimize gall formation of RKN by 58.8% and 70.7%. The combined treatment evidenced more inhibitory activity than any of the individual treatments [73]. Sharma and Sharma [60] found that beneficial microorganisms in two different groups, i.e., AMF and plant-growth-promoting rhizobacteria (PGPR), could have promising efficacy for controlling soil-borne diseases involving PPNs. The dual inoculation of mycorrhiza (*Rhizophagus irregularis*) and PGPR (*Pseudomonas jessenii* strain R62 and *Pseudomonas synxantha* strain R81) on tomato showed significant improvement in plant growth and diminished *M*. *incognita* infection. Further, this treatment displayed more activities of phenolics (28%) and defensive enzymes: peroxidase (1.26 fold), polyphenyloxidase (1.35 fold), and superoxide dismutase (1.09 fold). In contrast, a significant decrease in malondialdehyde (1.63 fold) and hydrogen peroxide (1.30 fold) content was recorded as compared to the untreated check (nematode-infected plants). Their greenhouse results proved the feasibility of AMF and PGPR singly or in combinations as potential BCAs for RKN management. Under field conditions, combined application of *Dactylaria brochopaga* (a nematode-trapping fungus) and *Verticilium chlamydosporium* (a nematode egg parasitic fungus) mixed with yeast molasses and vermiculate caused a 93.1% decrease in root galls per plant and also enhanced the weight of eggplant fruits per plant [74]. The nematicidal effect of rhizobacteria (species of *Pseudomonas* and *Serratia*) together or combined with *M*. *incognita* egg parasitic fungus *P*. *lilacinum* was better than that of their individual applications against this RKN [75]. Furthermore, these authors found that the combination of abamectin (produced during the fermentation process of the bacterium *Streptomyces avermitilis*) with *P*. *lilacinum* and the rhizobacteria was the most effective against *M*. *incognita*, followed by rhizobacteria and *P*. *lilacinum*. Their superiorities were recorded not only in terms of minimizing *M*. *incognita* galls and reproduction on plant roots but also in boosting plant growth parameters of tomato relative to the untreated check. So, they suggested that using these bioagents or their bioactive compounds like abamectin could be a potential antagonism strategy against PPNs in protected agriculture. In another study, the combined use of the fungus *Pochonia chlamydosporia* ZK7 and the bacterium *Bacillus nematocida* B16 could significantly boost *P*. *chlamydosporia* ZK7 activity to control *M*. *incognita*. Yet, because *B*. *nematocida* B16 singly did not manifest a distinct biocontrol efficacy, further studies revealed that B16 enabled more colonization of *P*. *chlamydosporia* ZK7 in the plant rhizosphere of tomato. This colonization was set on the account of *M*. *incognita* colonization ability [76]. Five materials, i.e., hexanal, furfural, (E)-2-hexenal, 2-nonanone, and benzaldehyde, were recorded to be highly shifted in the volatile compounds generated in the soil treated with this combination of bacterial and fungal application. Specifically, the shifts in benzaldehyde and 2-nonanone were the major factors that led to an enhanced colonization of *P*. *chlamydosporia* ZK7 in the tomato roots [76]. Thus, changes in chemical-signaling volatiles that are generated during the combined use of similar BCAs and that may result in increasing the biocontrol ability should be investigated to set novel PPN control strategies. Recently, combining *P*. *lilacinum* with avermectin was more effective than using either of them alone to boost *T*. *semipenetrans* control and protect citrus crops, offering a more sustainable regime for farmers [77]. Published books [27,71,78] reported other examples but there is still a need for improved PPN control.

Interestingly, many efficacious BCAs are versatile enough to serve in case of nematode disease complexes too [23,24,25]. The nematodes can initially predispose their sites of root penetration to other pathogens, enhance the susceptibility of their hosts by modulating physiology of host tissues, break host resistance to other fungal/bacterial pathogens, function as vectors of bacterial and viral pathogens, and alter the rhizosphere microflora. Thus, PPNs can typically synergize the wilt and root-rot-causing fungi [79] and bacteria [80], which result in developing serious disease complexes. Such synergism can be inflicted on key crops such as vegetables, cotton, legumes, tobacco, and others. Even a modest population of the fungal/bacterial pathogen can lead to a dangerous disease complex due to synergism induced by the PPNs [81,82,83,84,85]. Therefore, the magnitude of the interaction in these disease complexes must be detected and managed with durable techniques. Obviously, these techniques employ one of two main trends, using BCAs that have demonstrated big potential to suppress plant nematode population levels. One trend can function against root disease complex involving PPNs and other soil-borne pathogens, but the other trend can target PPNs alone. Any of these BCAs could be tested for the best integration with some of the aforementioned agricultural practices for either of the trends. Thus, recent assumptions are being researched and/or published. They should be thoroughly tested, developed, and documented for novel control strategies. Preferably, they comprise one or more BCAs that can be combined to perform two (or more) functions: boosting crop growth/yield and/or inhibit plant pathogenic fungi, bacteria, and/or nematode populations.

In this context, maximum improvement in plant growth and decrease in nematode–fungal infection parameters on mulberry in a field experiment was obtained via applying Furadan + Bio-consortium + Niger oil cake followed by Bio-consortium + Niger oil cake, without any significant difference between the two treatments [81]. The range in the improvement parameters of mulberry growth was as high as 77.7–87.7%. Moreover, the corresponding reduction in the severity of this wilt/root-knot complex disease engaging *M*. *incognita* with *Fuzarium solani* and *Botryodiplodia theobromae* was 88–93%. Additionally, these treatments achieved 93.8% plant survival over the check. So, the authors suggested the use of the Bio-consortium combined with Niger oil cake as an eco-friendly integrated disease management strategy for both managing root disease complex in mulberry and curbing leaf yield loss. For management of another root disease complex, synergistic interaction between *Radopholus similis* and *Phytophthora capsici* was quite apparent. It causes wilt complex and big yield loss of black pepper. Therefore, various BCAs (*Trichoderma harzianum*, *P*. *lilacinum*, *P*. *fluorescens*, and *Bacillus subtilis*) and other compounds (neem cake, Bordeaux mixture, and carbofuran) were evaluated against this wilt complex. The treatment comprising Bordeaux mixture combined with *P*. *lilacinum* significantly minimized the nematode population level and decreased foliar yellowing, defoliation of plants, and lesion indices of black pepper [82]. Saikia and Bhagawati [68] indicated that the treatment containing *Trichoderma viride*, *Glomus fasciculatum*, and mustard cake was highly beneficial in reducing the symptoms and losses of disease complex caused by *M*. *incognita* and *Rhizoctonia solani* and improving the growth of okra. Other investigations on disease complexes, especially those comprising root-rot pathogens and RKNs on different crops, have been documented and their biocontrol via effective IPM has been reported [83]. Further useful combined effects of BCAs with other components to prove their effectiveness against different PPN species on specific crops are given (Table 2). As a flush of such combinations is still in the pipeline, Table 2 presents one yearly example (2013 through 2025) to vividly express their counterparts that can further set large scales of novelty for safe and effective PPN control strategies.

In contrast, PPNs may interact with other microbial diseases but in beneficial ways too [97]. This may be evident, for example, in different mixed results when a plant is infected with a virus and becomes infected with a nematode too. While the root-knot index was higher on *Solanum khasium* roots inoculated with tobacco mosaic tobamovirus than on plants infected with the nematodes only [98], watermelon mosaic-polyvirus-infected zucchini (*Cucurbita pepo*) showed suppressing effects on *M*. *javanica* populations. The infection with the latter virus apparently retarded the establishment of *M*. *javanica* in the zucchini roots relative to healthy plants [99]. Such interactions, with positive or negative trends, took place or were clearer when PPN inoculation was preceded for 2–3 weeks by the virus infection. This implies definite biochemical reactions in plant tissues as crucial mechanisms were engaged in these processes [100]. Thus, the changes caused by one pathogen are either detrimental or favorable to the other. Interestingly, the influences of the host plants on these types of interaction were demonstrated on various crops [27,72,101]. Plant root exudation is a key pathway defining microbial communication with host and non-host plants. The exudates have an important role in selecting and enriching a specific set of microbial antagonists in the plant rhizosphere [29,30]. In other cases, exudates can be the primary defense line against plant invasion [19]. Additionally, some components of these exudates, like organic acids, amino acids, and secondary metabolites, can induce modifications in the PPN surface, which affect microbial attachment. Typically, a favorable interaction between plants and their beneficial root microbiota is linked to a low nematode performance on the host [30]. This is especially important in organic farming, where stimulating a favorable ecological equilibrium among soil biota without using synthetic pesticides is a priority. Ultimately, soil microbiome and plant genotypes could occasionally be manipulated in specific nematode-suppressive soils to conserve native biologicals that serve to control PPNs [16,30].

#### 2.2.4. Combined Measures Without BCAs for Management of PPNs

Other approaches, without BCAs, may still serve the same goal. Chemical nematicides are well known to be an effective control measure, but traditional compounds (oximecarbamates or organophosphate) are unlikely to be applied or developed if their toxicities are high. So, they should be cautiously used in conjunction with other tactics to diminish environmental impact and decrease the risk of resistance development, if applicable. Hence, currently unbanned nematicides can be utilized in combination with other techniques. Components such as crop rotation, tillage, mulching, solarization, and resistant crop varieties may be combined to boost efficacy and reduce negative impact. So, novel classes of nematicides with safe and different mechanisms of action are being sought [7,102,103]. Their perceptive design through bioinformatics approaches along with related genetics and biochemistry will aid in grasping such mechanisms to attain safe usage.

## 3. Prospects of Future Combinations

### 3.1. Astute Manipulation and Avoiding Drawbacks for Multi-Functional Management

Surely, while many of such combinations proved to be favorable (Table 2), there are also possible drawbacks of others. Such drawbacks may occur either between the applied BCAs, when combined with other components, or even between these other components, or combinations without BCAs. Antagonism between BCAs through competition and antibiosis has been reported to reduce their pesticidal performance in the greenhouse [104] and under field conditions [105]. Compared to the untreated control, individual treatments applied as seed coatings and seedling drenches of *Burkholderia cepacia* strains Bc-2 and Bc-F and of *Trichoderma virens* strain Gl-3 significantly reduced numbers of *M*. *incognita* eggs + J_2_ per g bell pepper root. Importantly, the egg + J_2_ numbers recorded from combining these treatments were not significantly different from untreated controls, suggesting that strain combinations decreased biocontrol effectiveness compared to applications of individual microbes [105]. Therefore, as there are numerous bio-nematicides which are or are likely to become widely available soon, the potential gains and defects of their combinations must be examined. Over and above, it is also quite possible that a single BCA may perform better than two other combined BCAs. In this regard, recently isolated strains of endophytic bacteria and fungi were tested against *M*. *incognita* on tomato plants [106]. These authors found that singly applied fungus *P. lilacinum* strain MR20 outperformed a mixture of two combined strains of plant-growth-promoting bacteria, *Pseudomonas fluorescens* MR12 and *Serratia marcescens* MR25, in suppressing the nematode population. The fungal strain MR20 showed higher effectiveness in decreasing the following nematode parameters: number of egg masses per root, number of J_2_s per 100 g soil, number of egg masses per 100 g soil, number of J_2_s + eggs of *M. incognita* per 100 g soil, reproduction factor, and their total reduction percentage after 60 days under greenhouse conditions.

Multidimensional control of multiple pathogens and/or pests should address the limitations and defects of IPM plans. Thus, a theoretical framework was recently put forward to control various pests/pathogens simultaneously [107]. Such a multi-target goal satisfies the practical needs of farmers who mostly face multiple pests and pathogens infesting single crops. The framework assures a holistic technique that incorporates systems thinking and multi-trophic interactions to further adopt “green” IPM strategies and tactics [108]. To this end, the future related combinations would be better designed with a multidimensional focus for better adoption with changing ecological conditions. This will necessitate examining the spatial dimension in terms of interaction of soil, pest, crop, introduced treatment, and natural enemy networks and temporal dimension, i.e., pest/pathogen-related interactions during the growing season too.

### 3.2. Examining Further Combinations on a Case-by-Case Basis to Enlighten Growers

It is suggested herein to examine the relevant effects of such sequential or simultaneous applications on a case-by-case basis. In this way, we can accurately determine the degree of compatibility among different ingredients in each combination and optimize PPN control according to the existing variables of each case. It would enable us to set and validate the factors needed to fulfill the best effective nematicidal combination [109]. While this technique can realize favorable matches for PPN control methods, it can join them to other control tactics for additional pests into IPM plans. These matches on a case-by-case basis should be considered in IPM. For example, although each of two strains of *Burkholderia cepacia* or *Trichoderma virens* strain Gl-3 could significantly reduce *M*. *incognita* numbers in terms of their eggs and J_2_ on roots of bell pepper, combining these BCAs failed to do so but, rather, they showed a nonsignificant difference compared with the untreated check [105]. Another example indicated that changing the timing of adding the same pesticides may change the interaction between them from additive to synergistic [110].

Following this strategy, we can satisfy the need for the adequate expertise of every individual disease or disease complex before initiating effective control measures. So, clear, specific, and actionable concluding results from relevant research would be highly beneficial. Subsequently, related agricultural extensions and various media tools should be a leading platform for disseminating these impactful combinations to stakeholders. They could help stimulate new directions in research through feedback and usage of promising combinations, something always needed. Their targets are to identify and widely disseminate settings under which the utilization of such combinations can act for cost-effective, value-added tactics and strategies to INM or even IPM. Conversely, if we find a lack of success stories in terms of deficiency in synergistic and additive combinations, it will signal the need for a deeper introspection within the relevant research priorities and BCAs.

### 3.3. Incorporation of Sophisticated Tools and Techniques for INM

Emerging techniques and advanced tools are offering promises of quite superior and novel strategies for PPN management. But how close are we to exploring and exploiting these technologies to meet demands for effective, inexpensive, and safe PPN control strategies? Surely, nematologists should harness emerging innovations to document and apply investigations and techniques such as the following on a large scale. Efficient PPN control strategies should initially set more effective quarantine measures to prevent the introduction of alien phytonematodes, slow down the rate of spread of recently introduced one(s), and/or limit their damage in order to spare time to develop alternative management strategies [111]. The polymerase chain reaction (PCR)-based technologies to accurately identify and quantify species of PPNs as well as biocontrol attributes of their related BCAs can contribute in these types of strategies. Campos-Herrera et al. [112] could design species-specific primers and TaqMan^®^ probes related to the ITS rDNA region of the entomopathogenic nematodes (EPNs). Thus, PCR-based techniques are useful for accurate detection of BCAs [112] or quarantine of PPNs [113] and support sampling, extraction, and identification methods for early detection of PPNs and BCAs before decision making [114].

Bioinformatics devices can easily and promptly chart the active ingredient interactions within biopesticides with their targets in order to tackle multifactorial diseases. Related key contributions via in silico methods are being developed [49,50]. A computer software package for the clustered, regularly interspaced short palindromic repeat (CRISPR) was set as a genome-aware platform to boost genetic assays on parasitic nematodes (CRISPR-PN2) [115]. The program offers flexible application and checks over the automated sketch of definite pilot RNA sequences for CRISPR experiments on PPNs. It helps in enabling high-throughput gene editing at the given scale. Thus, it serves for diversified BCAs with new favorable traits that are induced by mutation and/or genetic engineering. Obviously, such editing can also improve plants that do not have enough PPN-resistance sources.

It is well known that, on interacting with plant roots, BCAs generate various compounds like extracellular enzymes, toxins, secondary metabolites, and volatile organic compounds (VOCs) that suppress PPNs. The suppression may be indirectly attained by interaction between BCAs [76] or by guiding the root exudates during BCA colonization to enhance the secretion of RKN-repellent materials [116]. On the other hand, root exudates also play a key role in repelling pests/pathogens or attracting useful/harmful microorganisms toward plant roots [110,117]. The natural PPN surface coat could be valued as a new target for PPN management [118]. Other molecular studies have very recently revealed that *P*. *chlamydosporia* synergistically backs systemic plant defense response in *Phacelia tanacetifolia* against the RKN, *M*. *hapla* [118]. Hence, additional aspects of tripartite (plant–RKN–BCA/PGPR) interactions should be considered for incorporation in integrated management strategies [117,118]. The complex interaction between plants from one side and microbes or/and bioactive compounds on the other side in the rhizosphere against PPNs is mostly untapped. Exploring their interactions can open new avenues for detecting positive and negative interactions. Also, VOCs with olfactory signals proved to play commanding roles in communication and reaction of various organisms within their settings. Mounting interests in harnessing the full biocontrol spectrum toward safe and reliable PPN management could exploit these VOCs. Silva et al. [119] found that VOCs emitted from dry macerates of certain plant species reduced *M*. *incognita*-J_2_ motility to about 0% and minimized egg hatching by 47% relative to the untreated check. Moreover, VOCs from *Bertholletia excelsa* Humb. & Bonpl. (Ericales: Lecythidaceae) and *Brassica oleracea* Linnaeus (Brassicales: Brassicaceae) shoots killed J_2_ and reduced the number of galls and eggs in *M*.-*incognita*-infected tomato roots. Also, a gas chromatographer coupled to a mass spectrometer could chemically profile complex mixtures of plant volatiles in terms of essential oils (EOs). These EOs can mostly manifest multiple biological activities and interactions between their compounds regarding PPNs. So, Faria et al. [120] could profile the toxicity of eight EOs against *B*. *xylophilus* in comparison to their 1:1 mixtures in order to screen for favorable synergistic interactions. They could also characterize the main compounds of the best synergistic mixtures regarding their expected ecological fate and toxicity relative to emamectin benzoate, a standard nematicide for *B*. *xylophilus*. Basically, the nematicidal performance of EOs stems from their chemical groups, e.g., phenylpropanoids, terpenes, and organosulfur compounds. Among these, synergistic interactions against RKNs were identified between carvacrol and geraniol, as well as geraniol and eugenol. In contrast, binary combinations of carvacrol, γ-terpinene, and o-cymene exhibited decreased efficacy against RKNs [121]. Thus, grasping how such compounds interact can result in developing more reliable final products for nematode control.

A reliable technique to maintain BCA activity and longevity in adverse conditions is microencapsulation. Using this technique with multi-purpose BCA can strengthen IPM plans. *Trichoderma asperellum* is a good BCA of PPNs [41,122]. Also, in two field tests, soil solarization followed by applying alginate pellets based on *T*. *asperellum* led to the greatest reductions in strawberry black root rot incidence (59.3 and 74.1%) and severity (72.5 and 75.2%) caused by several fungal pathogens [123]. Nanoparticles (NPs) may introduce a revolution in managing key PPNs as well [124]. Mixing noninfectious viral NPs, by removing its RNA, with nematicide solutions can enable nematicide to penetrate the soil and kill the nematodes at comparatively deep plant root zones [125]. Additional merits of these NPs generally involve enhanced efficacy and fewer inputs of the required nematicides with consequently lower eco-toxicity. Nonetheless, more tests to assure NPs clean production and usage with cost-effective techniques are further warranted for risk-free and durable agriculture [126]. Within these contexts, PPN identification using multiple deep learning object detection models can contribute to generating sound datasets. These latter serve for quick propagation of such novel methodologies and provide a path towards broadly shared tools, results, and meta-analyses [127]. The models can ease multi-omics approaches that are being explored, tested, or applied to exploit untapped opportunities for PPN control. Molecular identification could be added to morphological and biochemical characters of PPNs and/or their BCAs to soundly and accurately detect novel species and strains. This is especially important as PCR-based methods could compare high-throughput genome sequencing (HTS) and real-time quantitative PCR (qPCR) for their merits in characterizing BCAs [128]. Using HTS, Dritsoulas et al. [129] developed fine-scale taxonomic resolution to differentiate closely related EPN species and consequently distinguish their specific phenotypes. Surely, these phenotypes—host specificity, reproductive potential, habitat adaptation, and persistence—are direly needed for fulfilling the best BCA–host matching. Ayaz et al. [130] reviewed multi-omics techniques involving metagenomics, transcriptomics, proteomics, and metabolomics, which have resulted in discovering nematicidal compounds. They stressed the potential importance of multi-omics approaches in biocontrol of PPNs to secure sustainable agriculture.

## 4. Broader Consideration of Combinations for IPM

Factually, focusing on merging two or more BCAs and/or agricultural practices/inputs for INM herein is intended to present and bring together the knowledge in applied nematology and emerging technologies. While INM refers to the state of the art [27], it is also important to resolve the current and future issues confronting IPM, including various pests and pathogens, not only PPNs. Hence, setting such merging for superior IPM and improved crop yields should continuously be explored and wisely exploited. Over and above, various methods and tools should be harnessed to achieve proper systems for their expansions. In the same direction, a progressive increase in tank mixing of agrochemicals and other agricultural inputs such as biopesticides is underway. Evolving practices are exemplified by timely mixing of agrochemicals with compatible biocontrol agent(s). As EPNs proved to function against insect and nematode pests [58,131], they may provide dual-purpose usage. When EPN *Steinernema glaseri* was added before chemical fertilizers, it showed better efficacy than *S*. *glaseri* that was tank-mixed with the chemicals simultaneously [132]. Consequently, field plots treated with *S*. *glaseri* that were not tank-mixed with chemicals led to high peanut germination rates close to those of chlorpyrifos (a chemical insecticide). Moreover, their results revealed that inorganic phosphorus fertilizer had more unfavorable effects than potassium and nitrogenous chemical fertilizers on *S*. *glaseri* virulence and consequent peanut germination. Therefore, Abd-Elgawad [133] stressed that timely application of tank mixing of BCAs like EPNs with other production practices/materials that can boost [134] biocontrol potential against host pests should be considered in the IPM context. Otherwise, such a potential is reduced [135]. Obviously, the order and way in which one adds the relevant components into a tank can lower incompatibility issues. Hence, it is essential to consider key attributes of the used BCAs as well as chemical characteristics and compatibility of mixtures at different application rates in various IPM schemes. For instance, *Pasteuria* spp. are well-known bacterial nematicides with effective mechanisms on key nematode species (Table 1). Their endospores are resistant to chemical destruction, mechanical shearing, and desiccation. Additionally, they function as most heat-resistant forms under field conditions and possess a prolonged shelf life too. With such merits, they could also be utilized in a tank-mixed tactic with several other nematicides/insecticides or mixed with seeds a few days before cultivation to reduce crop losses and aid in evolving reliable and safe pest management [136]. Moreover, these endospores can better function with microelements, organic/soil amendments, and inorganic fertilizers so that they can act synergistically or additively with other agricultural inputs. Therefore, a tank mix of several inputs, involving a species of *Pasteuria*, would likely be more significant in terms of saving money, effort, and time [43]. Currently, Clariva Elite Beans™ was released as a seed treatment product that has nematicidal, insecticidal, and fungicidal effects. Its active ingredients are *Pasteuria nishizawae*-Pn1, sedaxane, thiamethoxam, fludioxonil, and mefenoxam [136]. Practically, *Pasteuria* spp. can also be used not simultaneously but in rotation with other production inputs to delay/limit the development of PPN resistance. This would be conducted to avoid the selective pressure on the pest imposed by a unique mechanism of a single agricultural exercise. Also, the natural role of earthworms like *Eisenia fetida* against PPNs should be exploited. Integrating these earthworm products could provide a nutrient supply for the host plant while controlling the RKN (*M. javanica*) within the concept of organic food production [137]. On the other hand, Li et al. [138] highlighted the functional significance of three vascular bundle development-related genes in cucumber against *M*. *incognita*, offering insights into developing plant defense mechanisms.

Such techniques, based on conducting simultaneous or sequential applications, should be operated on a wider scale/globally to achieve better gains for using promising BCAs like *Pasteuria* spp. With merits such as their obligate parasitic nature and biodiversity, these bacteria can offer key and efficient biocontrol combinations. Recently, it was confirmed that their soil-added endospores can rapidly launch and deliver season-long performance and function under relatively harsh ecological conditions and soil pH [43]. Meanwhile, *Pasteuria* spp. do not disturb nontarget organisms. Therefore, they can be integrated with other materials and/or techniques to manage PPNs in organic farms via the best bacterium–host matching. They may be engaged in various sustainable schemes via boosting other related inputs such as crop rotation, cover crops, and PPN-resistant cultivars. Likewise, *Bacillus* genus contains favorable species for PPN control. When its spores are introduced to the cultivated land, they may promptly initiate season-long activity and act under relatively severe environmental conditions and variable soil moisture, temperature, and pH. They are also endorsed for integration with other cultural inputs to optimize their control efficacy [45]. Such integration can widen the biocontrol spectra of many BCAs to other pest groups like those of organic farming and other pests infecting low-value crops. Definitely, other aspects for their successful use engage optimizing BCA delivery and persistence, priming them/their bioactive components, and reshaping their biocontrol for superior transformative impact [139].

Tank mix components can involve some of various chemical pesticides (herbicides, nematicides, fungicides, acaricides, insecticides, and bactericides), products containing biologicals (biopesticides, plant growth promoters, plant strengtheners, and soil conditioners), and other products (fertilizers, biostimulants, and adjuvants). Nonetheless, such components should be used strictly according to their legal product labels. An exception from legality reported by FIFRA [140] allows for mixing any pesticides or fertilizer(s) and pesticide(s) only if the mix is not explicitly forbidden by the product label. In addition to avoiding legal incompatibility, chemical and physical ones should also be deemed. Physical incompatibility happens when the components in the mixture do not mix well. It may include sedimentation, flocculation, phase separation, oil separation, shaping lumps, and formation of crystals, cream, and/or foam [141]. Jar tests and following the ammonium sulfate; powder solubles; powder dry; liquid flowables; emulsifiable concentrates; solutions (A.P.P.L.E.S.) mixing order can block most physical incompatibilities [142]. Chemical incompatibility takes place when a chemical reaction between tank mix components renders the active ingredient(s) deactivated. Signals of this incompatibility may not be apparent during mixing. Therefore, chemical incompatibility may not be recognized until after the treatment has been applied. In contrast, some of these incompatibilities may be identified by an elevated solution temperature showing an exothermic reaction. The heat can occasionally cause defects in the spray equipment. Factors such as pH and electrical conductivity should be considered to avoid chemical incompatibility. Also, such types of incompatibility can occur as a positively charged ingredient (cation) binds to a negatively charged ingredient (anion) [141,142,143,144]. Adopting such principles for IPM, tank mix can offer a variety of benefits engaging elevated pest control, a wider pest control spectrum (several target species), decreased selection pressure for resistance on using multiple modes of action, and optimized efficiency by consolidating multi-functional applications into one [143]. It leads to better management by minimizing costs, efforts, entry into the used site, and soil compaction [143]. Also, it is worth mentioning that challenges in developing reduced-risk agricultural nematicides that are more selective, less toxic, and safer to use were recently reviewed [144].

## 5. Conclusions

Contrary to many effective but unhealthy synthetic chemical nematicides, safe bionematicides are frequently less effective/consistent and/or slower-acting in nematode control. So, the reliability of bionematicides or even other PPN control measures could be mostly boosted via their combination. Such a simultaneous or sequential combination should focus on their additive or synergistic effects while considering their multi-functionality and metabolic complementarity. Therefore, the end in view is to absorb and wisely manipulate all factors engaged in their interactions to obtain soundly formulated, benign, and reliable pesticides. These factors would be sifted in two dimensions, spatial and temporal. The spatial dimension focuses on interaction of soil, pest, crop, introduced treatment, and other existing fauna and flora networks. The temporal dimension deals with pest/pathogen-related interactions during the whole target crop- growing season. Thus, grasping interactions, behavior, and sensitivities of the constituents in any INM/IPM plan under the existing conditions is essential to optimized pest management. It offers better adoption of control measures in terms of shifting environmental conditions and effectiveness against several pathogens/pests along the growing seasons of the intended crops. While challenges remain in adopting sufficient related commercial products, their cost-effective formulation, ease of registration, and relevant farmer awareness should be earnestly sought. In contrast to these ready-made products, ensuring legality and pesticide compatibility with components in tank mixes should be assured. Moreover, to overcome restrictions related to this optimization, it is mandatory to incorporate advanced techniques like nanopesticides, microencapsulation, bioinformatics, volatile organic compounds as signals for soil inhabitants, and RNA-Seq in pesticide development. Promising outputs of such techniques should be applied on large scales in IPM strategies to provide a comprehensive approach for raising crop yields and fostering agricultural sustainability.

## Figures and Tables

**Table 1:** Fungal and bacterial nematicides and their modes of action on specific targets *.

Fungi and Bacteria	Nematode (PPN) Target/Host Crop(s)	Mode of Action	Ref.
1. Fungi: *Aspergillus niger* van Tieghem (Eurotiales: Aspergillaceae)	*Meloidogyne incognita*/mung bean. *Meloidogyne* spp./tomato. *M. javanica*/pigeonpea and chickpea. *M. incognita*/okra and brinjal. *Heterodera glycines*/soybean	Egg parasite and induces systemic resistance against PPNs. The fungus contacts with a cyst or an egg mass to grow/colonize it, rapidly blocking larval formation. Its effective nematicidal metabolite is a calcium oxalate co-ordination compound	[37,38]
*Purpureocillium lilacinum* (Thom) Luangsa-ard, Hou- braken, Hywel-Jones & Samson (Hypocreales: Ophiocordycipitaceae)	*Meloidogyne graminicola*/rice. *M. incognita*/black gram, bitter gourd, brinjal, potato, cowpea, betelvine, chrysanthemum, banana, cardamom, okra, tobacco, mock orange. *M. javanica*/tomato, tobacco. *Rotylenchulus reniformis*/tomato, chickpea. *Heterodera cajani*/pigeonpea. *H*. *avenae*/barley. *Radopholus similis*/banana. *Tylenchulus semipenetrans*/citrus, jambhiri, Khasi Mandarin. *M. arenaria*/brinjal	Egg parasite and produces antibiotics, viz., leucinostatin and lilacin and enzymes such as protease and chitinase, degrading the eggshell/inhibiting hatch. The degraded chitin releases ammonia, toxic to J2 of root-knot nematodes (RKNs). Its hyphae enter the vulva/anus of RKN females. The fungus penetrates the egg and develops profusely in and over the eggs, inhibiting juvenile development. The eggs swell/buckle. The developing juvenile in the egg is broken by the hyphae. Many conidiophores are produced and the hypha moves to the adjacent eggs	[39]
*Pochonia chlamydosporia* (Goddard) Zare & W. Gams (Hypocreales: Clavicipitaceae)	*M*. *incognita*/tomato, bell pepper, brinjal, pigeonpea, common bean, lettuce. *M. javanica*/tomato, pepper, lettuce, broad bean, okra. *H*. *schachtii*/sugar beet. *H. cajani*/pigeonpea. *H. avenae*/wheat. *M*. *hapla*/tomato, lettuce. *R. reniformis*/cotton	Egg/adult female parasite of PPNs. The root-knot and cyst nematodes are the main hosts. It parasitizes citrus, burrowing, and reniform nematodes. It can colonize roots of plant species and induce local resistance in fungal–nematode–plant interactions with differential ability of two out of five isolates to induce systemic resistance against *M. incognita* in tomato but not in cucumber	[40]
*Trichoderma harzianum* Rifai (Hypocreales: Hypocreaceae)	*M. javanica*/tomato. *H. cajani*/pigeonpea. *M. incognita*/chickpea, pea, brinjal, cardamom, French bean, pigeonpea, green gram, tomato. *M. arenaria*/maize. *H. cajani*/pigeonpea. *M. graminicola*/paddy	Secretes many lytic enzymes like chitinase, glucanases, and proteases which help parasitism of PPN eggs. The chitin layer is dissolved through enzymatic activity. The hyphae of *T*. *harzianum* penetrate the eggs and juvenile cuticle, proliferate within the organism, and produce toxic metabolites	[41]
*Trichoderma viride* Pers. (Hypocreales: Hypocreaceae)	*M. incognita*/okra, tomato, green gram, betelvine, mulberry, soybean, chickpea, cowpea, cucumber, wheat. *M. javanica*/tomato, mungbean, brinjal. *Helicotylenchus multicinctus*/banana. *Pratyl-enchus thornei*/chickpea. *M. graminicola*/rice. *R. reniformis*/cowpea	Produces antibiotics like trichodermim, dermadin, trichoviridin, and sesquiterpene heptalic acid, which are involved in the suppression of nematodes and inhibiting egg hatching. Plant defense induction/priming via enhanced root development (nematode resistance and stress resistance)	[42]
2. Bacteria: *Pasteuria* spp. (Bacillales: Pasteuriace-ae): *P*. *penetrans* Sayre & Starr. *P*. *nishizawae* Sayre & Starr (Sayre et al.). *P*. *thornei* Starr & Sayre. *P*. *usgae* Giblin-Davis et al.	Various nematode species of *Meloidogyne*, *Pratylenchus*, *Heterodera*, *Globodera*, *Hoplolaimus* and *Belonolaimus* on many vegetables, soybean, cotton, cucurbits, and floriculture.	Bacterial spores are attached to the nematode’s body and germinate, forming a germ tube that penetrates the body cuticle. Vegetative mycelial colonies eventually fill the body with a large number of endospores, leading to death or at least to a reduction in nematode feeding and reproduction	[43]
*Pseudomonas fluorescens* Flugge (Migula) (Pseudomonadales: Pseudomonadaceae)	*M. graminicola*/rice. *M. incognita*/field pea, okra, tomato, brinjal, mulberry, grapevine, cucumber, sugarbeet, black gram, jasmine. *H. cajani*/pigeonpea. *R. similis*/banana. *Hirschmanniella gracilis*/rice. *P*. *thornei*/chickpea. *T. semipenetrans*/citrus	Produce antibiotics, viz., phenazines, tropolone, pyrrolnitrin, pyocyanin, and 2,4- diacetylphloro-glucinol, which have a suppressive effect on plant parasitic nematodes. The efficiency of the induction capacity of the defense system in different plants by inducers depends on the plant species	[44]
*Bacillus* spp. (Bacillales: Bacillaceae): *B*. *firmus* Bredemann & Werner. *B*. *thuringiensis* Berliner. *B*. *subtilis* Ehrenberg (Cohn)	*M*. *incognita*, *Radopholus similis*, *Ditylenchus dipsaci*, *Xiphinema index*, *Heterodera* sp., *T*. *semipenetrans* and *Meloidogyne* spp. on many vegetables and field crops, as well as ornamentals (e.g., tomato, strawberries, flowers, trees, vines, permanent and seasonal crops)	Their mechanisms against the nematodes may vary from one species to another but they generally embrace antibiotic production, antagonism, and/or induced resistance. Specifically, their activities for RKN control depend on cry proteins–toxic particles of *Bacillus thuringiensis*, toxic antibiotic production, and expelling second-stage juveniles of RKNs by *B*. *cereus*; genes encode surfactin and iturin synthesis as antibiotics by *B*. *subtilis* to suppress *Meloidogyne* spp. populations, enzymatic activity of *B*. *firmus* that is able to colonize the plant roots; these bacteria can protect the roots and antagonize the nematodes	[45]
*Streptomyces* spp. (Streptomycetales: Streptomycetaceae): *S*. *avermitilis* Kim & Goodfellow. *S*. *yatensis* Saintpierre et al. *S*. *pactum* Bhuyan et al. *S*. *rochei* Berger et al. *S*. *lincolnensis* Mason et al. *S*. *antibioticus* (Waksman & Woodruff) Waksman & Henrici	*Meloidogyne* spp. especially *M*. *incognita*, *M*. *javanica* and *M*. *arenaria* on many crops like tomato, rice, pepper, cabbage, peanut, soybean, mungbean, watermelon, cucumber	They produce secondary metabolites that exhibit potent nematicidal properties. Arenimycin, carboxamycin, fervenulin, hygromycin, and lincomycin are some of the Streptomyces-derived compounds that proved to have nematicidal potential. These bacteria also act as an elicitor of plant defense against nematode intruders. They evinced endophytic potential, plant growth promotion mechanism, compatible nature with other antagonists, and were safe to nontarget organisms	[46]

* Updated and reconstructed after Abd-Elgawad and Askary [25]. The biotic/abiotic factors that induced the nematicidal efficacy and related mechanisms are detailed in the cited references (Ref.).

**Table 2:** Combined effect of biocontrol agents (BCAs) or resistant genotypes with other components in integrated nematode management.

BCA	Integrated with	The Target	Crop	Efficacy	Ref.
*Pasteuria penetrans*	Castor cake	*Meloidogyne incognita*	Chilli	Reduced galling index (84.75%) and *M*. *incognita* soil population (85.74%)	[84]
*T. viride*	*Pochonia chlamy-dosporia* + Urea	*M. incognita*	Kidney bean	Significantly reduced galls and egg masses per root system	[85]
*P. chlamydosporia*	Neem cake	*M. javanica*	Brinjal	A significant reduction in *M. javanica* reproduction when neem cake or mustard cake was added to the fungus-treated soil	[86]
	Mustard cake				
*Syncephalastrum racemosum*	*Paecilomyces lilacinus*	*M. incognita*	Cucumber	Their combination significantly decreased the nematode density so severely that it is proposed as a new biocontrol strategy	[87]
*P. chlamydosporia*	Neem cake	*H. zeae*	Sweet corn	Decline in cyst population in soil by 54.35%	[88]
*Bacillus megaterium*	nemastrol	*Meloidogyne* spp.	Sugar beet	Integration of two or more components of such bio-agents gave better results in plant growth parameters than did single ones. The best suppression in nematode population (95.7%), root galling (83.0%), and egg masses (100%) was obtained by nemastrol + humic acid + bio-arc + sweet basil callus + oxamyl	[89]
	humic acid				
	dried sweet basil callus				
*Bacillus licheniformis*	*Pseudomonas aeruginosa*	*M. incognita*	Tomato/eggplant	Their combination was highly effective in suppressing root knot reproduction but increasing plant growth parameters	[90]
Nematode-resistant tomato cultivars	Fumigation with chloropicrin	*Meloidogyne* spp.	Tomato	Soil fumigation significantly increased fruit yield, especially in fall (95%) and to a lesser extent in spring (14.5%). Resistant cultivar Sanibel produced the highest fruit yields	[91]
*Pseudomonas dimunita*	3 isolates of *Bacillus* sp.	*Meloidogyne* spp.	Tomato	In a fortified formulation, they increased fruit weight by 33.61% and suppressed the total nematode populations.	[92]
*Trichoderma harzianum*	1,3 dichloropropene and organic matter	*M. incognita*	Tomato	Their combination caused synergistic effects of both plant growth/yield and *M. incognita* control relative to individual usage	[93]
*Bacillus Para-licheniformi FMCH001*	*B. subtilis FMCH002*	*Meloidogyne* spp.	Tomato/Soybean	The combination interfered to reduce population densities of different stages of the RKNs and others (i.e., *Pratylenchus* spp.) via multiple modes of action	[94]
*Bacillus velezensis* VB7	*Trichoderma* *koningiopsis* TK	*Meloidogyne* spp.	Tomato	Increased diversity and abundance of rhizosphere bacterial communities that might be responsible for enhanced nematicidal properties. Their combined applications can enhance the nematicidal action to curb RKN infecting tomatoes	[95]
*Colletotrichum nigrum*	*Lantana* extract +fluopyram	*Meloidogyne* spp.	Tomato	Significantly reduced nematode number (>21%) and gall (<2.0) and egg mass (<1.5) indices, and increased shoot height (>80%), dry weight (>70%), and dry root weight (>50%)	[96]
